# Effects of Medical Cannabis Treatment for Autistic Children on Family Accommodation: An Open-Label Mixed-Methods Study

**DOI:** 10.3390/children12101373

**Published:** 2025-10-11

**Authors:** Ayelet David, Eynat Gal, Ayelet Ben-Sasson, Elkana Kohn, Matitiahu Berkovitch, Orit Stolar

**Affiliations:** 1 Department of Occupational Therapy, Faculty of Social Welfare & Health Sciences, University of Haifa, Haifa 3490002, Israel; 2 Clinical Pharmacology and Toxicology, Shamir Medical Center (Assaf Harofeh), Zerifin 6093000, Israel; 3 Clinical Pharmacology and Toxicology, Tel-Aviv University, Tel-Aviv 69978, Israel; 4 Child Development Centers, Sharon District-Maccabi Healthcare Services, Kfar Saba 4465141, Israel

**Keywords:** autism, family accommodation, parental well-being, CBD-rich cannabis

## Abstract

**Highlights:**

**What are the main findings?**

- CBD-rich cannabis treatment over 6 months was associated with reduced family accommodation (FA) and parental distress in families of autistic children.
- Qualitative findings showed improved family routines, parental well-being, and greater engagement in meaningful activities and social interactions.

**Implications**

- CBD-rich cannabis treatment may reduce FA and parental distress, while improving family routines and well-being.
- These results provide preliminary support for CBD-rich cannabis treatment in autistic children, though further controlled studies are needed.

**Abstract:**

Background/Objectives: Parents of autistic children often face behavioral and participation challenges of their children, leading them to make accommodations to maintain a stable daily family routine. These family accommodations (FA) involve adapting family routines, actively engaging with the child’s support needs and symptoms, and avoiding specific situations. Methods: This open-label, mixed-methods study investigated the impact of CBD-rich cannabis treatment on FA. In the quantitative phase, analyses included 44 parents (from 87 initially recruited) who had complete FAS-RRB data at baseline, 3 months, and 6 months. In the following qualitative phase, 15 parents from the full sample participated in semi-structured interviews. Results: Quantitative results showed reductions in FA frequency and parental distress at 3 and 6 months. Qualitative findings revealed positive changes in family routines, enhanced well-being, and improved parental engagement in meaningful activities and social interactions. Conclusions: This study provides preliminary evidence that CBD-rich cannabis treatment may reduce family accommodation (FA) and parental distress, while improving family routines and well-being. However, given the open-label design and observed adverse events and withdrawals, the findings should be interpreted with caution.

## 1. Introduction

Autism spectrum disorder (ASD) is a pervasive clinically and etiologically heterogeneous neurodevelopmental condition. Its diagnostic criteria in the Diagnostic and Statistical Manual of Mental Disorders (5th ed.; DSM-5) include differences in social communication and restricted and repetitive behaviors (RRBI) [1]. Many autistic children have co-occurring maladaptive behaviors like aggression, tantrums, impulsiveness, sleep problems, and mealtime challenges [2] and diagnoses such as attention-deficit/hyperactivity disorder and anxiety [3].

These common behaviors and diagnoses have been associated with decreased daily functioning and participation of children with autism [4,5,6]. Consequently, they increase parental stress and adversely affect family participation and quality of life [7]. Despite various available interventions for autistic children, parents report that their children continue to experience serious challenges that influence the child and the entire family unit. Presentations vary widely across individuals and are influenced by context factors such as sleep, sensory environment, and daily routines.

Although the cannabis plant has been used medicinally since ancient times, only in the past decade has a more comprehensive understanding of its structure enabled extensive research into its potential health benefits [8]. Previous research distinguished two main phytocannabinoids: cannabidiol (CBD) and tetrahydrocannabinol (THC) [9,10]. The primary psychoactive substance in the plant, THC, is responsible for the “high” sensation, whereas CBD is an antioxidant, anti-inflammatory substance with antipsychotic and anti-anxiety properties [11,12]. By isolating these cannabinoids, medical cannabis can be tailored to treat specific medical needs. In the past decade, the FDA approved using CBD-rich cannabis to treat children with specific types of refractory epilepsy [13]. Interestingly, parents of autistic children treated for epilepsy with CBD-rich cannabis noticed improved ASD symptoms [14]. Recent studies showed the effectiveness of CBD-rich cannabis treatment for many conditions and symptoms common in ASD [15]. Nevertheless, previous studies also reported side effects and treatment withdrawals; these issues are discussed below.

These findings led to more research that found CBD-rich cannabis treatment effective and safe for autistic children [16,17,18,19]. Specifically, studies reported improved communication and socialization skills [16,18,19,20] and participation in daily activities like sleeping [18,21] and eating [22]. Furthermore, studies reported that cannabis consumption reduced co-occurring symptoms, including self-injury, tantrums, restlessness, and anxiety [16,17,18,20]. In our recently published study [23], we observed significant reductions in RRBI, particularly in compulsive, ritualistic, and sameness behaviors, alongside notable improvements in overall anxiety and specific subtypes, such as general, social, panic, and separation anxieties, following 6 months of CBD-rich cannabis treatment in autistic children. Our findings suggest that reductions in anxiety, especially in panic- and separation-related subtypes, predicted subsequent decreases in RRBI, specifically in sameness behaviors.

However, studies have also highlighted that CBD-rich cannabis treatment is not without limitations, including common side effects. These adverse effects include changes in appetite, gastrointestinal symptoms, irritability, somnolence, psychoactive effects, and sleep disturbances [16,17,18,19,21,23,24]. A recent study found that while 75% of parents reported only mild side effects and were able to adhere to the treatment, 9% of participants experienced severe side effects leading to treatment withdrawal. Additional reasons for withdrawal included challenges with the treatment regimen and, in some cases, unrealistic parental expectations, which complicated adherence for certain families [25].

These findings emphasize the need for professional guidance to help parents of autistic children understand both the benefits and potential challenges of CBD-rich cannabis treatment for their children and themselves. A recent study by Mazza et al. [26] further suggests that the benefits of CBD treatment extend beyond clinical and medical outcomes, positively impacting not only the child’s quality of life but also that of their families.

Family accommodation (FA) is a collection of the family’s responses or adjustments to the daily life demands of a child with a disability, intended to minimize the stress associated with the disorder and sustain the family’s daily routine [27,28]. These responses may include modifying family routines, actively participating in the disorder symptoms, or avoiding situations that might trigger symptoms associated with the syndrome [28].

Studies indicated that FA is prevalent among families of children with obsessive-compulsive disorder (OCD) [29,30], anxiety [31,32], sensory modulation disorders [33,34,35], and ASD [27,36,37,38]. They also associated FA with adverse clinical presentations, including more severe symptoms, poorer treatment outcomes, increased parental burden, and more disrupted family functioning [28].

Increased interest motivated the development of several scales to measure FA associated with anxiety, OCD [39], and sensory over-responsivity [33]. Feldman et al. [27] adapted the Family Accommodation Scale to measure RRBI accommodation for the ASD population. They found that 80% of parents of autistic children engage in FA at least monthly, and 55% engage daily. Studies linked higher FA frequency in ASD to higher RRBI appearance [27,38], more disruptive behaviors [40], poorer communication and daily living skills [27], increased difficulty tolerating uncertainty [36], and increased child anxiety [31].

Although these studies emphasized how the high FA prevalence interferes with child presentation, they inevitably referred to the parents’ role within this process. Beyond the child’s behaviors, studies correlating FA with parenting stress [40] recently demonstrated that both the child’s and the parents’ anxiety contribute to increased FA [36].

While parents perform FA to relieve stress, most parents report feeling distress due to the necessary accommodation—and sometimes link this distress with their children’s aggression when not accommodated [27]. Although these accommodations may contribute to the family members’ well-being and daily functioning—and indeed help in the short term—they can become maladaptive over time, disrupting the children’s participation in their environments and even causing more severe symptoms [28,32].

Given the association of FA with a broad array of child and family challenges attributed to ASD, it is a crucial target outcome for intervention. Intervention research examining FA outcomes with autistic children has been scarce. The related literature includes studies of autism and co-occurring anxiety disorders [31,41] and of autistic adolescents and adults and co-occurring OCD [42,43]. These studies associated FA with treatment outcomes: Russell et al. [42] found that higher FA levels predicted poorer treatment outcomes, and Storch et al. [31] found significantly reduced FA with the child’s reduced anxiety. A more recent study found that parent-led cognitive behavioral therapy supported the parents’ ability to manage their child’s anxiety without accommodating it [44].

The literature supports the positive effects of medical cannabis treatment for autistic children. However, no research has yet examined its impact on FA. Hence, the main goals of this mixed-methods study were to (1) examine the impact of medical cannabis treatment on parent-reported FA and (2) deepen the understanding of parents’ perspectives on changes in FA following cannabis treatment.

Although prior psychosocial interventions have reported changes in FA, no cannabis studies to date have included FA as an outcome. This study directly addresses that gap.

## 2. Materials and Methods

Primary and secondary outcomes: The pre-specified primary outcome was the frequency of family accommodation (FAS-RRB items 1–7 total score). Secondary outcomes were the child’s short-term response when no accommodation was provided (FAS-RRB items 9–11) and parental distress related to accommodation (FAS-RRB item 8). Participant flow. In total, 87 families were recruited; 65 children completed the 6-month CBD-rich protocol; of these, 44 parents provided complete FAS-RRB data at baseline, 3 months, and 6 months and were included in the primary analyses.

### 2.1. Procedure and Participants

**Figure 1:**
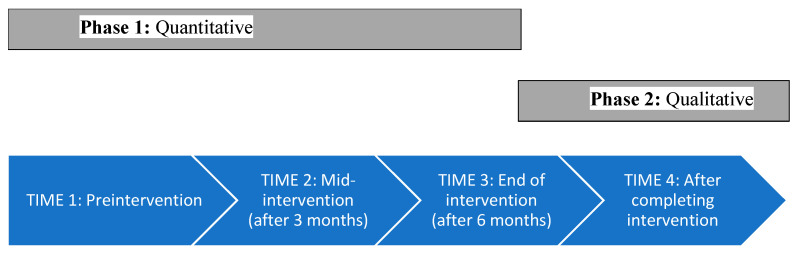
Sequential Mixed-Methods Research Design.

We used an explanatory, sequential mixed-methods design [45,46] in which the quantitative trends explicitly informed the qualitative interview guide; integration was planned a priori via connecting (building the interview topics on quantitative patterns) and weaving the two strands to produce meta-inferences. The quantitative study related to the impact on FA of medical cannabis treatment for autistic children. The follow-up qualitative phenomenological study included in-depth interviews to deepen understanding and contextualize the phenomena [47], as shown in Figure 1.

### 2.2. Phase 1: Quantitative Study

We conducted the quantitative research as part of an open-label study at Shamir Medical Center in Israel, recruiting participants through social media platforms and in collaboration with the Israeli Society for Autistic Children. Inclusion criteria were children aged 5 to 12 years with a medical diagnosis of ASD (based on *DSM-5* criteria) accepted by the Ministry of Health. We confirmed the ASD diagnosis using the Autism Diagnostic Observation Schedule™ [48]. Furthermore, parental reports of their children’s disruptive behavioral problems (e.g., violence, restlessness, and sleep problems) during the last 6 months were an inclusion criterion. Exclusion criteria were children with known genetic syndromes causing autism symptoms or diagnosed metabolic diseases and parents with mental health problems (e.g., psychosis or drug addiction). No IQ prerequisites were set for Phase 1.

A pediatric neurologist specializing in ASD interviewed the participants’ parents, and concomitant medication regimens that were in place before enrollment were expected to remain stable throughout the study. The cannabis treatment protocol was individualized for each patient using a personalized medicine approach. Participants received medical cannabis extract infused in medium chain triglyceride oil with a 20:1 CBD:THC ratio (Nitzan Spectrum^®^, Seach Medical Group, Jerusalem, Israel) for 6 months. This ratio was chosen based on open-label studies that found it safe and effective for autistic children [14,17,24]. Titration followed a pragmatic schedule starting at one drop/day (5.7 mg CBD, 0.3 mg THC per drop) and increased gradually as clinically indicated based on parents’ reports of tolerability and target behaviors; the final dose did not exceed 10 mg/kg/day CBD (maximum 400 mg/day) and 0.5 mg/kg/day THC (maximum 20 mg/day). Dose timing was individualized according to parental feedback (e.g., higher doses at night to support sleep). Across participants, total daily doses averaged 2.87 mg/kg CBD (SD ≈ 1.22); observed ranges were 0.42–6.67 mg/kg CBD and 0.02–0.35 mg/kg THC.

Biweekly follow-up interviews conducted by study staff recorded comorbid symptoms, safety, and adherence; clinic visits occurred at baseline (T1), 3 months (T2), and 6 months (T3). Parent-reported adverse events were categorized (e.g., appetite change, gastrointestinal symptoms, somnolence, irritability, psychoactive effects, sleep disturbances). Parents completed the Family Accommodation Scale for Restricted and Repetitive Behaviors (FAS-RRB) [27] three times: before the medical cannabis treatment (Time 1), after 3 months of treatment (Time 2), and after 6 months of treatment (Time 3). They also completed the demographic data questionnaire at Time 1.

**Figure 2:**
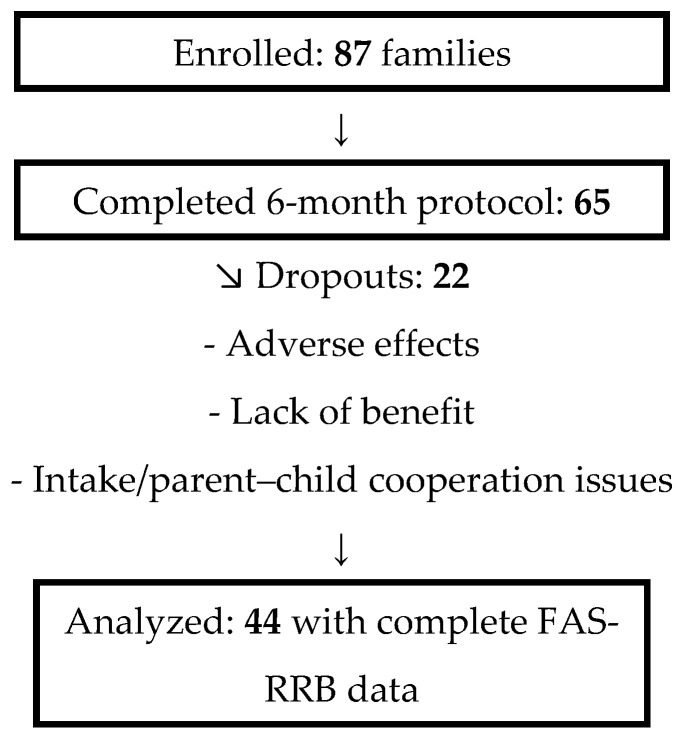
Participant flow.

A total of 87 parents of autistic children (ages 5–12 years; M = 7.39, SD = 2.02; 71 boys [81.6%], 16 girls [18.4%]) were initially enrolled. Of these, 65 (74.7%) children completed the 6-month CBD-rich cannabis treatment. Twenty-two (25.3%) dropped out before completion, for reasons including adverse effects such as worsened functioning (n = 2), violence (n = 3), abdominal pain (n = 1), weight gain (n = 1), and sleep problems (n = 1); parental perception that the treatment was ineffective (n = 9); and intake difficulties or parent–child cooperation issues (n = 5) [23]. Among the completers, 44 provided complete FAS-RRB data at all three time points, forming the analytic sample. Participant flow is summarized in Figure 2.

### 2.3. Phase 2: Qualitative, Time 4

Over the 3 months following the medical cannabis treatment, an occupational therapist conducted semi-structured interviews with 15 parents selected through purposeful sampling from the full sample of the quantitative study. Inclusion criteria were (a) participation in the former study, (b) completion of the 6 months of CBD-rich cannabis treatment, and (c) language fluency. We further recommended that the parent who was most involved in the child’s eating and sleeping times participate in the interview. As a result, only mothers were interviewed. We acknowledge that interviewing mothers only limits representativeness of broader family perspectives.

Parents who consented to participate in the qualitative stage were interviewed online and audio-recorded using secure university Zoom software (premium version). The interviews lasted approximately 45 min each and were transcribed after deleting all identifying details. Internal consistency for the FAS-RRB has been reported as high in autistic samples; to our knowledge, minimal important change/responsiveness have not yet been established, so we report effect sizes and exact *p*-values to aid interpretation.

### 2.4. Measures

#### 2.4.1. Medical Demographic Questionnaire

This questionnaire included medical and demographic questions regarding variables such as gender, age, co-occurring diagnoses, and medications.

#### 2.4.2. Family Accommodation Scale for Restricted and Repetitive Behaviors

The FAS-RRB [27] is an adapted version of a scale used in FA studies on OCD and anxiety disorders [30]. It assesses the frequency of accommodating behaviors that families of autistic children experience due to RRBI (e.g., “Have you changed your work habits because of your child’s repetitive behaviors?”). The scale includes 11 items rated on a 5-point Likert scale from 0 (never) to 4 (daily). The first seven items relate to the frequency of accommodating behaviors and are summed to a general score from 0 to 28; higher scores indicate that more FA is needed. The eighth item addresses parental distress caused by accommodating behaviors (e.g., “Did the assistance you gave the child in these forms cause you distress?”). The last three items relate to the child’s short-term response if no parental accommodation is made (e.g., “Did the child respond aggressively when you did not help him?”). Internal consistency was high for the seven FAS-RRB items (α = 0.93) and the entire 11-item scale (α = 0.85).

### 2.5. Interviews

Parents were asked open-ended questions within a semi-structured interview guide. Topics included (a) their overall experience with the treatment for themselves and their family, (b) the changes they perceived following the medical cannabis treatment, and (c) the implications for their personal and family lives and routines. They were encouraged to discuss both benefits and challenges, while the interviewer explored these issues in depth, seeking specific examples, emotions, and dilemmas the parents faced during the intervention process.

### 2.6. Data Analysis

The study population’s demographic characteristics were described using descriptive statistics. We conducted a repeated measures analysis of variance (ANOVA) followed by a post hoc Bonferroni test to determine which periods differed significantly. The aim was to examine the cannabis treatment effects on the frequency of accommodating behaviors and the children’s short-term responses. Due to the abnormal distribution of the sample, we used Friedman tests to examine the treatment’s effects on parental distress and Wilcoxon signed rank tests to determine whether the differences between time-period frequencies were significant.

Three experienced occupational therapists analyzed the transcribed interviews in a three-stage systematic phenomenological approach [49]: (1) Identify meaningful text units into thematic codes, (2) group and map the codes into six categories that demonstrate commonalities and differences, and (3) merge the categories into three conceptualized major themes [50]. Each author independently coded the first three interviews into preliminary categories to map the remaining interviews and stopped interviewing upon reaching data saturation after the 15th participant [51].

Data trustworthiness was achieved by presenting rich citations from the original text (with encrypted locations), documenting detailed descriptions of the process, and journaling thoughts and feelings reflected during the interviews to identify potential bias [50]. Alongside a peer review of the data, the authors—supported by their familiarity with the phenomena and existing literature—conducted open conceptual discussions until they reached agreement.

## 3. Results

### 3.1. Quantitative

#### 3.1.1. Descriptive Statistics

Forty-four parents of the autistic children who completed the 6-month cannabis treatment (34 boys and 10 girls; 5–12 years, M = 7.39 years, SD = 2.14) completed the FAS-RRB at all three time points. Of those parents, 40 (91%) were married and living together, and 14 (31.8%) reported that their child used prescribed medications: sleep remedies (71%), stimulants (21%), and antipsychotics (14%).

#### 3.1.2. Family Accommodation Frequencies

We applied the repeated measures ANOVA followed by Bonferroni adjustments to avoid the possibility of alpha-error inflation. Wilk’s lambda (Λ = 0.649), F(4, 170) = 10.25, *p* < 0.001, and ηp2 = 0.194 indicated a difference in the FAS-RRB scores following treatment. The following univariate test demonstrated differences in accommodating behavior frequency and the children’s short-term responses. Table 1 lists the outcome measures’ means, standard deviations, *p* values, and effect sizes (ηp2) from the RM univariate test.

The post hoc Bonferroni test results revealed that the mean scores for accommodating-behavior frequency and the child’s short-term response subscales were significantly higher at Time 1 (pretreatment) than at Times 2 and 3 (posttreatment). That is, FA and the children’s maladaptive behavior decreased if their parents made no accommodations following the cannabis treatment. This decrease stabilized as the intervention continued: We found no significant differences between the Times 2 and 3 mean scores in either subscale.

The Friedman test indicated a significant difference between the three times in the parental distress subscale (n = 44, χ2(2) = 7.56, *p* = 0.023, Λ = 0.085). Specifically, the Wilcoxon signed rank tests showed a significant difference based on positive ranks between Time 1 (Mdn = 2) and Time 2 (Mdn = 1), Z = −3.003, *p* = 0.003, r = 0.452. Parental distress decreased following the cannabis treatment when no parental accommodation was made. However, we found no significant difference between the mean scores at Times 2 and 3. Thus, the apparent rebound at 6 months relative to 3 months was not statistically significant and is unlikely to be clinically meaningful as the median remained 1 at both time points.

### 3.2. Qualitative (Themes)

The qualitative analysis highlighted the parents’ feelings of relief due to their children’s behavioral improvements and reduced need for FA, as well as the subsequent effects on the parents’ daily lives. The phenomenological analysis of the in-depth interviews revealed three key themes (summarized in Table 2): changes in the parents’ sense of well-being, parents’ ability to find and maintain meaningful employment or leisure activities, and the overall family environment.

#### 3.2.1. Parental Sense of Well-Being

Most (12/15) of the mothers consistently described a substantial change in the family’s sense of well-being. Specifically, they described decreases in their autistic children’s maladaptive behaviors, which they associated with the children’s ability to be “more attentive,” “calmer,” and “more present” following the cannabis treatment. They described these changes as reducing the need for FA. Nevertheless, a minority (3/15) described limited or inconsistent benefits; see Theme 3 for details.

Y (mother of a 9-year-old) shared the positive change in her overall sense of well-being due to her 9-year-old son’s ability to reduce his need for her constant touch to calm down. His changed needs reduced the FA, which manifested in symptom-related actions:

*He really needed my touch. To relax, he needed my hug all the time. He used to say, “I need to calm down. Hug me now. I need to breathe, give me a hand.” … In the last year [posttreatment], I could go rest, and he would not bother me.*

N (mother of an 11-year-old) elaborated on her changed stress level and reduced need for alertness due to changes in her son’s ability to participate in daily routine and family activities, such as praying with his father. She reported a reduced need for her FA, like avoiding activities that could trigger her son’s stress: “You can be more relaxed. You can enjoy your food… Once these behaviors have decreased, it allows me as a human being to enjoy the situation a little bit more and not be so alert.” N emphasized that the changes in her son’s maladaptive behavior were significant but not a cure:

*In the course of the intervention, I felt [the maladaptive behavior] had diminished—not perfectly at all, but in a significant way … Something about him was positively affected. If he feels well, then we are all well.*

The interviews revealed that most of the family’s day before the cannabis treatment revolved around the autistic child’s needs and the required accommodations. Parents described their daily lives as unpredictable, with great difficulty maintaining routines for themselves or for their family. They mentioned frequent FA to maintain daily routines primarily for sleeping, eating, and transitioning between contexts and activities.

S (mother of an 11-year-old) elaborated on the extreme symptom-related accommodation her family used during mealtime to address her 11-year-old autistic son’s needs and demands:

*He’s sitting down to eat in the living room [or] kitchen. We [the family] had to shut ourselves in a different room. Everyone needed to prepare the food and leave “his” area … Cannabis decreased the anxieties, so [now] he can eat next to us, which is a huge improvement for us. We don’t have to run away from home anymore.*

Through her words, S demonstrated positive changes in the family’s routine and sense of well-being following her child’s decreased anxiety during the cannabis treatment.

Most (9/15) mothers mentioned the positive effects of this treatment on the child’s ability to fall and stay asleep. The most commonly mentioned pretreatment FA was sitting with the child for long hours at night, which was incompatible with the child’s age. According to the mothers, these symptom-related FAs disturbed their quality of life. L (mother of a 7-year-old) explained, “You don’t sleep at night for 3 months, and you have to be constantly awake and be with him… And then we started cannabis, and, really, it was a huge improvement.” D added that the cannabis contributed to her marriage due to her son’s earlier bedtime: “The fact that he sleeps early [following the cannabis] is a lot, a lot. It gives me and my husband some time together.” The child’s improved ability to fall asleep and maintain sleep during the cannabis treatment positively affected the parents’ well-being.

The mothers also described their FA as avoiding, delaying, or performing actions to maintain a daily routine, sometimes at the expense of the other family members. All noted that much of their pretreatment days had been occupied with their autistic child’s needs; the other family members were expected to understand and support the mother. D, a mother of five, described becoming more available for her autistic son’s four younger siblings due to his changed ability to understand situations and be more independent after cannabis treatment:

*The little siblings said, “Mom, why are you always just with K? Only K! He’s bigger and can do it alone. We’re small, and we need your help.” And today [after the cannabis], it’s less, like there’s a change. Today, he understands. He can play by himself; he can be alone. And then I can also spend time with my little kids and help them.*

#### 3.2.2. Parents’ Ability to Find and Maintain Meaningful Occupations/Jobs

A third (5/15) of the mothers shared how their child’s decreased maladaptive behaviors and improved daily participation reduced their need for FA and enhanced their ability to engage in meaningful preferred activities, such as an occupation/job—besides merely addressing their children’s needs. N elaborated on her son’s improved emotional regulation, which allowed her to plan her day. She explained how her son’s mood used to determine her day—whether she could go to work—and the stress around that uncertainty:

*One day, he wakes up OK, and one day, he doesn’t. I could go to sleep and think, “Tomorrow, I need to do X, Y, Z,” but I could not know how the morning would look … I don’t have this problem anymore; I don’t feel this stress.*

L shared the FA her family used to do to support her son’s sleep difficulties. It did not allow her to wake up in the mornings or focus on other activities because she was too exhausted: “Before [cannabis], I could plan with you and after not sleeping at night, I didn’t show up. I couldn’t. I could not come. I was too tired.”

Both N and L reported relief that they could engage in meaningful occupations/jobs following the intervention. L added that she could even attend business meetings in the evenings—she knew the evening routine would be fine without her presence: “Today, I’m more relaxed, first of all, to bring someone to look after the kids, and I know it’s going to be OK. I now work in the mornings, and in the evenings, I actually go to events!” Employment and activity changes were self-reported by mothers; no external verification (e.g., employment records) was collected.

These examples demonstrate how the mothers were less preoccupied with the autistic child’s needs posttreatment. Some even asked for outsourcing support. The families’ routines improved as their stress and alertness for unexpected events decreased.

#### 3.2.3. Parent and Family Environment

Throughout the interviews, most (10/15) participants discussed their difficulty leaving the safe home and engaging in out-of-home activities. The mothers shared with pain the family’s price tag for avoiding overwhelming stimulation and crowded places for the child’s sake. They especially mentioned refraining from family activities such as going on vacations or to restaurants, hosting family and friends, and being hosted by others. Following the intervention, most (12/15) mothers indicated positive changes in their children’s ability to self-regulate and participate in social settings. These positive changes in the child’s presentation decreased the need to avoid family outings. D described: Reports distinguished between tolerance of crowded public places (e.g., restaurants) and participation in structured family outings (e.g., visiting relatives), with improvements noted in both.

*Before [cannabis], he wouldn’t stay where there were too many people. He would refuse to sit down. He would always inspect whether it suited him … where to sit, where not to sit … Today, there’s no problem. Today, you go out, sit, it doesn’t matter where. It doesn’t matter if there’s a lot of people*. No additional follow-up beyond the 6-month treatment period was conducted; therefore, persistence of these changes beyond this window was not assessed.

Consistent with the quantitative variability, one fifth (3/15) of the mothers reported that the cannabis had no beneficial effect on the need for FA. R, the mother of a 5-year-old autistic child, described the treatment’s inconsistent effects on her child: “If I focus on the effect of the cannabis oil, the change was on and off. That is what was so hard. One day, you could be calm; one day, it feels like I haven’t given anything.” Indeed, an inconsistent response from the child was described as not only affecting the FA consistency but also affecting the mothers’ general sense of well-being. Mothers who reported inconsistent effects did not identify specific patterns (e.g., time-of-day or dose-related triggers).

## 4. Discussion

As medical cannabis is increasingly used to treat autistic children, recent studies have primarily focused on examining its effects on child outcomes [13,23,52]. However, studies examining the effects of this treatment on the families of autistic children remain scarce.

The current mixed-methods study examined the effects of a 6-month medical cannabis treatment for autistic children on FA and parental well-being. Its results support the potential effectiveness of this treatment in reducing FA frequency and parental distress. Furthermore, the findings highlight mostly positive changes in the family’s overall well-being, as well as the parents’ ability to find and maintain meaningful occupations and engage in social interactions following the child’s treatment. Given the open-label design of this study, the findings should be viewed as associations rather than causal effects, reflecting parent-reported perceptions.

These findings align with a recent study [26], which found that the benefits of cannabis treatment for autistic children extended beyond the clinical and medical scope, leading to an overall improvement in both the patient’s and the family’s quality of life.

Specifically, our results indicate a significant decrease in FA at 3 months (Time 2) and 6 months (Time 3) of treatment. However, the decrease between Time 2 and Time 3 was not significant. Our in-depth interviews further supported this decrease in FA posttreatment and shed light on its positive effects. The mothers appreciated being able to spend quality time with their other children and spouses. They emphasized their renewed ability to have family meals, go on family outings, and enjoy simple daily activities as a family. They also highlighted the positive impact of their enhanced ability to engage in meaningful social and professional activities.

Earlier studies demonstrated reductions in FA following other interventions for children and youth with autism. For example, Storch et al.’s [31] and Frank et al.’s [41] intervention studies with children and youth with ASD and anxiety showed that following cognitive behavioral therapy, FA decreased significantly from pre- to postintervention. These studies align with our findings, supporting the notion that interventions targeting the child’s symptoms also positively affect parental factors like FA.

Of substantial importance in addition to decreased FA, our quantitative results suggest decreased parental distress after 3 months of cannabis treatment. The parents related this reduction to their children’s lessened maladaptive behaviors, in turn decreasing the parents’ need to accommodate constantly.

Unexpectedly, the decreased parental distress observed after 3 months of treatment (Time 2) was not sustained after 3 more months (Time 3). The opposite was true: Parental distress reemerged, although not to levels as high as at Time 1. These results may allude to a ceiling in the child’s behavioral improvements following cannabis treatment, a habituation effect in the parents’ response, or a placebo/expectation effect—reflected in parents’ hope and excitement for a promising treatment with few or no side effects compared with previous medicines. In an open-label study, statistical regression toward the mean is also plausible and could contribute to the observed pattern.

In our study, 25.3% of participants discontinued treatment, and among those who completed the 6-month protocol, some parents reported no meaningful change in FA, as reflected in the quantitative results. Our recently published study [25] similarly found that unrealistic parental expectations and hopes pose significant adherence challenges. That work emphasized that CBD-rich cannabis treatment is not without barriers, underscoring the need for professional guidance and education for parents of autistic children regarding its potential impact. Further longitudinal research is warranted to examine whether the observed changes in child behaviors and parental accommodations can be sustained over the long term (i.e., years of treatment) and whether they persist once treatment ends.

Our findings further highlight the bidirectional effects that FA has on parents. On the one hand, the mothers elaborated on the intense necessity to satisfy their children’s needs and support their participation in even basic daily activities. On the other hand, they mentioned the intense energy required to provide these accommodations. They described the hardship of the accommodating effort and the stressful effects of such accommodations on their relationships with their other children, spouses, work, leisure, and emotional well-being. These hardships suggest the crucial importance of reducing FA, as mentioned in other studies [38,40].

Previous studies have addressed this bidirectional effect and suggest that, although necessary, FA may create a vicious cycle by worsening the child’s symptoms over time: The children increasingly rely on these accommodations, and their maladaptive behaviors may escalate when the FA are not provided [28,32]. Our study’s quantitative results also show a significant decrease in the children’s negative short-term response after 3 months of cannabis treatment when parents do not provide FA. These results might indicate the treatment’s indirect role in breaking that vicious behavioral cycle by reducing the need for FA. To clarify mechanisms, future work should prospectively assess potential mediators (e.g., anxiety or sleep) that may link treatment-related reductions in FA with the child’s reduced short-term negative response.

Recent research has associated higher FA levels at the beginning of intervention with poorer intervention outcomes for youth with autism and anxiety [41,53]. Based on the results of the current and previous studies, we suggest that identifying elevated FA levels at the intervention’s assessment stage may be of enhanced importance and that reduced FA could be an outcome for interventions with autistic children. Including FA when evaluating intervention outcomes would provide a more comprehensive and integrated approach to assessing the interventions’ impacts on children and parents/families.

### 4.1. Limitations

This study has several limitations that warrant consideration. First, owing to the open-label design, neither participants nor outcome assessors were blinded, and outcomes relied primarily on parent-reported measures; together these factors could inflate perceived benefits via expectancy or placebo effects. Accordingly, randomized, double-blind, placebo-controlled trials are needed to more rigorously establish efficacy.

Second, individualized dose titration and concomitant medications complicate attribution of change and obscure potential dose–response relationships.

Third, the qualitative sample comprised only mothers and was conducted within a specific cultural and health-system context, which may limit generalizability.

Fourth, COVID-19 restrictions resulted in missing follow-up questionnaires for some parents. Although 65 participants completed the treatment protocol, only 44 had complete FAS-RRB assessments at all three time points. Analyzing only complete-case data (n = 44) may introduce bias; future studies should consider multiple imputation and prespecified sensitivity analyses to address missingness.

### 4.2. Implications

The results of this study indicate positive—yet preliminary—implications for medical cannabis on child behavior and extend understanding of its potential effects on family/parental factors among families of autistic children. The findings also support routinely tracking family accommodation (FA) as a clinically meaningful outcome alongside parental distress and the child’s short-term response when FA is withheld. Given variability across families and co-treatments, these implications should be applied cautiously and with sensitivity to context.

## Figures and Tables

**Table 1:** Means and Standard Deviations of Outcome Measures and *p* Values and Effect Sizes (η2) of RM-Univariate Test Showing Differences in Accommodating Behavior Frequency and Child’s Short-Term Response and Means and Standard Deviations of Friedman Test of Outcome Measures Showing Differences in Parental Distress.

Outcome		M (SD)		F(df = 2.86)	*p*	ηp2
	Time 1 n = 44	Time 2 n = 44	Time 3 n = 44			
Accommodating behavior frequency	16.45 (7.79)	12.34 (7.57)	12.22 (7.71)	13.58	<0.001	0.24
Child’s short-term response	7.95 (3.30)	5.22 (3.70)	5.29 (3.81)	19.13	<0.001	0.31
Parental distress	2.04 (1.72) Mdn = 2	1.20 (1.40) Mdn = 1	1.613 (1.46) Mdn = 1	χ2(2) = 7.56	0.023	r = 0.45

Note. FAS-RRB scoring: FA frequency (items 1–7) summed 0–28, higher = more accommodation; child short-term response (items 9–11) summed 0–12, higher = more negative response when no accommodation is provided; parental distress (item 8) scored 0–4, higher = greater distress. Effect sizes are partial η^2^ (~0.01 small, ~0.06 medium, ≥0.14 large). Pairwise post hoc tests used Bonferroni adjustment.

**Table 2:** Summary of Manual Coding Categories.

Theme	Coding Category
1. Parental sense of well-being *“You can be more relaxed. You can enjoy your food.”*	Reduced parental stress and decreased need for constant alertness due to fewer maladaptive behaviors of the child Consistent maintenance of daily routines, such as sleeping and eating; increased availability for other family members, including the spouse and siblings
2. Parents’ ability to find and maintain meaningful occupations/jobs *“I couldn’t wake up for work; I was just tired… Today, I work in the mornings, and, in the evening, I actually go to events.”*	Ability to plan the day in advance Confidence that the child will be fine without the mother’s presence
3. Parent and family environment *“[Preintervention,] we could not go to other family members that he didn’t want to visit. Cannabis gave him more support; he felt more confident.”*	Child’s improved ability to participate in social settings Increased ability to leave the safe home and engage in out-of-home family activities

## Data Availability

The data presented in this study are not publicly available due to ethical approval guidelines. The data can be requested from the corresponding author.

## Abbreviations

The following abbreviations are used in this manuscript:

ANOVA	Analysis of variance
ASD	Autism spectrum disorder
CBD	Cannabidiol
*DSM-5*	*Diagnostic and Statistical Manual of Mental Disorders* (5th ed)
FA	family accommodations
FAS-RRB	Family Accommodation Scale for Restricted and Repetitive Behaviors
OCD	Obsessive–compulsive disorder
RRBI	Restricted and repetitive behaviors
THC	Tetrahydrocannabinol
